# Supplementary material for: Hepatitis C Core-Antigen Testing from Dried Blood Spots
Source: Viruses. 2019 Sep 6;11(9):830. doi: 10.3390/v11090830 (PMC6784259; doi:10.3390/v11090830)
Supplement: Supplementary file 1 [file viruses-11-00830-s001.pdf]

Title: Hepatitis C core-antigen testing from dried blood spots

Authors:

Mia J. Biondi[1,2,3*], Marjolein van Tilborg[1,4*], David Smookler[1,2], Gregory Heymann [1], Analiza Aquino[5], Stephen Perusini[6], Erin Mandel[1,2], Robert A. Kozak[7], Vera Cherepanov[1], Matthew Kowgier[8], Bettina Hansen[1,2], Lee W. Goneau[6], Harry L.A. Janssen[1,2], Tony Mazzulli[5,6], Gavin Cloherty[9], Robert J. de Knegt[4], Jordan J. Feld[1,2,10]

SUPPLIMENTAL FIGURES AND TABLES

Supplementary Figure 1. Correlation between serum core-Ag and HCV-RNA from serum.

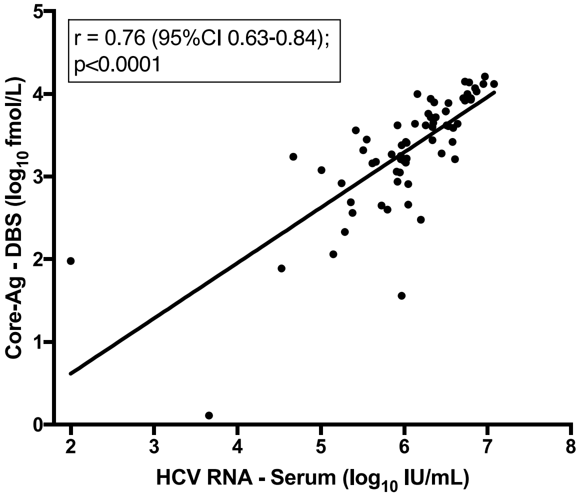

Supplementary Table 1. Cohort characteristics.

Baseline variable	n=117
Gender	
Male	69 (59.0%)
Female	48 (41.0%)
Age	55.8 (± 11.7)
HCV Genotype	
1a	44 (37.6%)
1b	26 (22.2%)
1 (unspecified)	7 (6.0%)
2	9 (7.7%)
3	21 (17.9%)
4	4 (3.4%)
5	0
6	2 (1.7%)
Mixed genotype	3 (2.6%)
Unknown	1 (0.9%)
Co-infection	
HBV	2 (1.7%)
Cirrhosis	
Yes	33 (28.2%)
No	84 (71.8%)
Treatment status	
Naïve	89 (76.1%)
Non-responder/treatment relapse	24 (20.5%)
Discontinued due to adverse events	4 (3.4%)

Supplementary Table 2. Concordance between DBS duplicates per condition and cut-off.

Test	-80ºC	+4ºC	+21ºC	+37ºC	+37ºC/+4ºC
HCV-Ab	67/68 (99%)	67/68 (99%)	67/68 (99%)	68/68 (100%)	66/68 (97%)
Core-Ag (Agreement > or <3 fmol/L)	67/68 (99%)	65/68 (96%)	66/68 (97%)	65/68 (96%)	66/68 (97%)
Core-Ag (Agreement > or <10 fmol/L)	66/68 (97%)	64/68 (94%)	66/68 (97%)	63/68 (93%)	66/68 (97%)

Supplementary Table 3. Sensitivity and specificity of HCV core antigen from DBS.

Sample Type: DBS Number	HCV Core-Ag Qualitative	HCV Core-Ag Quantitative
	**Sensitivity >3fmol/L (95% CI)** n=49	**Sensitivity >10fmol/L (95% CI)** n=49
**Venous:1**	91.8% (84.2-99.5%)	87.8% (79.0-97.0%)
**Venous:2**	93.9% (87.2-100%)	87.8% (79.0-97.0%)
**Finger-prick:1**	91.8% (84.2-99.5%)	81.6% (70.8-92.5%)
**Finger-prick:1**	93.9% (87.2-100%)	85.7% (75.9-95.5%)
